# Regional Neurodegeneration *in vitro*: The Protective Role of Neural Activity

**DOI:** 10.3389/fncom.2021.580107

**Published:** 2021-03-29

**Authors:** Rosalind E. Mott, Catherine R. von Reyn, Bonnie L. Firestein, David F. Meaney

**Affiliations:** ^1^Cell Press, Cambridge, MA, United States; ^2^School of Biomedical Engineering, Science and Health Systems, Drexel University, Philadelphia, PA, United States; ^3^Department of Neurobiology and Anatomy, Drexel University College of Medicine, Philadelphia, PA, United States; ^4^Department of Cell Biology and Neuroscience, Rutgers, The State University of New Jersey, Piscataway, NJ, United States; ^5^Department of Bioengineering, University of Pennsylvania, Philadelphia, PA, United States; ^6^Department of Neurosurgery, University of Pennsylvania, Philadelphia, PA, United States

**Keywords:** traumatic brain injury, neural networks, repair, microtrauma, recovery

## Abstract

Traumatic brain injury is a devastating public health problem, the eighth leading cause of death across the world. To improve our understanding of how injury at the cellular scale affects neural circuit function, we developed a protocol to precisely injure individual neurons within an *in vitro* neural network. We used high speed calcium imaging to estimate alterations in neural activity and connectivity that occur followed targeted microtrauma. Our studies show that mechanically injured neurons inactivate following microtrauma and eventually re-integrate into the network. Single neuron re-integration is dependent on its activity prior to injury and initial connections in the network: more active and integrated neurons are more resistant to microtrauma and more likely to re-integrate into the network. Micromechanical injury leads to neuronal death 6 h post-injury in a subset of both injured and uninjured neurons. Interestingly, neural activity and network participation after injury were associated with survival in linear discriminate analysis (77.3% correct prediction, Wilks' Lambda = 0.838). Based on this observation, we modulated neuronal activity to rescue neurons after microtrauma. Inhibition of neuronal activity provided much greater survivability than did activation of neurons (ANOVA, *p* < 0.01 with *post-hoc* Tukey HSD, *p* < 0.01). Rescue of neurons by blocking activity in the post-acute period is partially mediated by mitochondrial energetics, as we observed silencing neurons after micromechanical injury led to a significant reduction in mitochondrial calcium accumulation. Overall, the present study provides deeper insight into the propagation of injury within networks, demonstrating that together the initial activity, network structure, and post-injury activity levels contribute to the progressive changes in a neural circuit after mechanical trauma.

## Introduction

Traumatic brain injury (TBI) is a devastating public health problem with ~1.7 million estimated cases and 50,000 deaths each year (Faul et al., 2010). TBI can result from and display a collection of primary injuries and complications, including contusions, hematomas, diffuse axonal injury, cerebral ischemia, and brain swelling (Graham et al., 2006). Treatment options are limited in preventing the poorly understood secondary phase of injury, which often includes necrosis, apoptotic cell death, progressive axonal injury, oxidative damage, and changes in ionic homeostasis (Algattas and Huang, 2014). Ultimately, the neuropathological sequelae can result in debilitating cognitive deficits that persist for months to years.

The complexity of TBI is well-represented by *in vivo* models, but understanding the basic mechanisms of the “mechano-pathology” requires simplified *in vitro* models. The most common of these models utilizes substrate deformation to stretch cells to levels observed *in vivo* on a millisecond scale (Zhang et al., 1996; Geddes and Cargill, 2001; Lusardi et al., 2004). Alternatively, a fluid shear stress model can be used to injure cells plated on a solid surface or within a deformable matrix (LaPlaca and Thibault, 1998; Cullen and LaPlaca, 2006). Additional models have investigated mechanisms of diffuse axonal injury (DAI) by straining the axon population along a principal axis (Wolf et al., 2001) which can be more clearly defined with micro-patterning techniques (Tang-Schomer et al., 2010; Omelchenko et al., 2019). For local injuries, a number of techniques have been employed, including those that are initiated with membrane cutting with needles or scalpels (Mukhin et al., 1997, 1998; Lööv et al., 2012). To avoid membrane tearing, but to include deformation, a micro-injection device has been effectively used to rapidly pulse fluid onto axon bundles resulting in their deformation (Chung et al., 2005; Staal et al., 2010).

Despite the large number of technical approaches to model TBI *in vitro*, we know little about the relative threshold needed for mechanical injury to affect *network* function. As damage to neurons inevitably affects the subsequent recovery of a network, a detailed study is needed to understand the limits, or tolerance, of the network to physical force. Rather than solely focusing on the mechanisms that regulate the loss of neurons within the network, relating damage of nodes in the network to the spontaneous activity *and recovery* within microcircuits will yield critical functional thresholds for networks to mechanical damage. Moreover, the pattern of damage at the micro-scale may strongly influence the emergence of activity patterns at the macroscopic scale in TBI, a scientific area that is receiving more focus in the clinical imaging studies (Nakamura et al., 2009; Bonnelle et al., 2011; Caeyenberghs et al., 2012, 2014; Ham and Sharp, 2012; Palacios et al., 2013; Sharp et al., 2014; Fagerholm et al., 2015; Venkatesan et al., 2015).

To more specifically examine the relationship between precise injury to neurons and network function, we designed a microfluidics-based tool to precisely injure individual neurons within a living microcircuit. We used time lapse calcium imaging to estimate neural activity and functional network connectivity, linking injury to individual neurons to the activity of neighboring cells. We found that injuring a greater fraction of neurons within a network progressively impaired the activity and structure of the network. Similarly, we found that neuronal survival was influenced by the initial neuronal activity and functional connectivity of a neuron within the network. Blocking neuronal activity was more effective than either activating the network or allowing the network to naturally recover its activity, and blocking neural activity correlated with reduction of calcium uptake into the mitochondria, one mechanism of neuronal degeneration after trauma.

## Materials and Methods

### Cell Culture

All animal procedures were completed in accordance with the University of Pennsylvania Institutional Animal Care and Use Committee. All materials were obtained from Invitrogen Corporation (Carlsbad, CA) unless otherwise noted. Embryos at day E18 were surgically removed from a timed pregnant Sprague-Dawley rat anesthetized with 5% CO_2_ and sacrificed via cervical dislocation. Neocortical tissue was dissected from the embryos and dissociated for 15 min at 37°C in trypsin (1.4 mg/ml) and DNAse (0.6 mg/ml, Roche Applied Science, Indianapolis, IN). After trituration and filtration through Nitex mesh (Crosswire Cloth, Bellmawr, NJ), cells were resuspended in Minimum Essential Medium (MEM) with Earl's salts and GlutaMAX™ supplemented with 0.6% D-glucose (Sigma-Aldrich, St. Louis, MO), 1% Pen-Strep, and 10% Horse Serum and grown on glass bottom dishes (MatTek, Ashland, MA) coated with poly-D-lysine (0.08 mg/ml, Sigma-Aldrich) and laminin (0.001 mg/ml, BD Biosciences, San Jose, CA). After overnight adhesion, the plating medium was removed, and cultures were maintained in Neurobasal™ medium supplemented with B-27 and 0.4 mM GlutaMAX™ until maturity (14–19 days *in vitro* (DIV) for injury validation studies or 18–22 DIV for network studies). In a subset of studies, cultures were treated with the following drugs (Sigma-Aldrich, unless otherwise specified): APV (2-amino-5-phosphonovaleric acid) at 50 μM, BMB [(-)-bicuculline methbromide] at 50 μM, and NBQX (2,3-Dihydroxy-6-nitro-7-sulphamoylbenzo[f]-quinoxaline, Calbiochem, La Jolla, CA) at 50–500 nM, or TTX (tetrodotoxin) at 1 μM. Excitatory tone in the neural networks was determined by a previously published method in which BMB and APV were added to allow excitatory AMPAR connections to drive synchronized activity (Breskin et al., 2006). The incremental addition of NBQX eventually blocked the oscillatory activity at a concentration reported as [NBQX]/K_d_, where K_d_ = 47 nM (Patel et al., 2012).

### Micropipette Production and Calibration

Thin walled borosilicate capillary tubes (Sutter# BF150-110-10) were pulled with a Flaming/Brown style micropipette puller (Sutter, Novato, CA) to generate a short taper (3–4 mm) 1–2 μm tip. To obtain larger tips, the micropipettes were guided with a micromanipulator (Eppendorf, Hamburg, Germany) and tapped back to ~5 μm by depressing onto a glass surface. Micropipettes were filled with extracellular saline solution and connected to a FemtoJet device (Eppendorf) set to a known input pressure. The output pressure from the tip was dependent upon the total resistance to fluid flow of the micropipette and connective tubing and was determined by two calibration methods. A force calibration method using micro-array post detectors (mPADs) to estimate the pressure field applied to a stationary but deformable cell was developed. In this method, microfabricated arrays of silicone elastomeric posts deflect when nanonewton levels of force are applied (Sniadecki and Chen, 2007). The tip of the micropipette was positioned at a measured distance and height away from an array of posts. Using the FemtoJet, a 1 s pressure pulse deflected the post array during image acquisition at 50 frames per second. The images were analyzed in Matlab (MathWorks, Natick, MA) with an algorithm written to compute the centroid values of each post and to generate a displacement field from the data. The displacement field was used to compute the force field applied to the mPAD substrate, and the cross-sectional area of the cylindrical post was used to calculate a pressure estimate (Supplementary Information 1).

### Calibration and Measurement of Pulse Field

**Figure 1:**
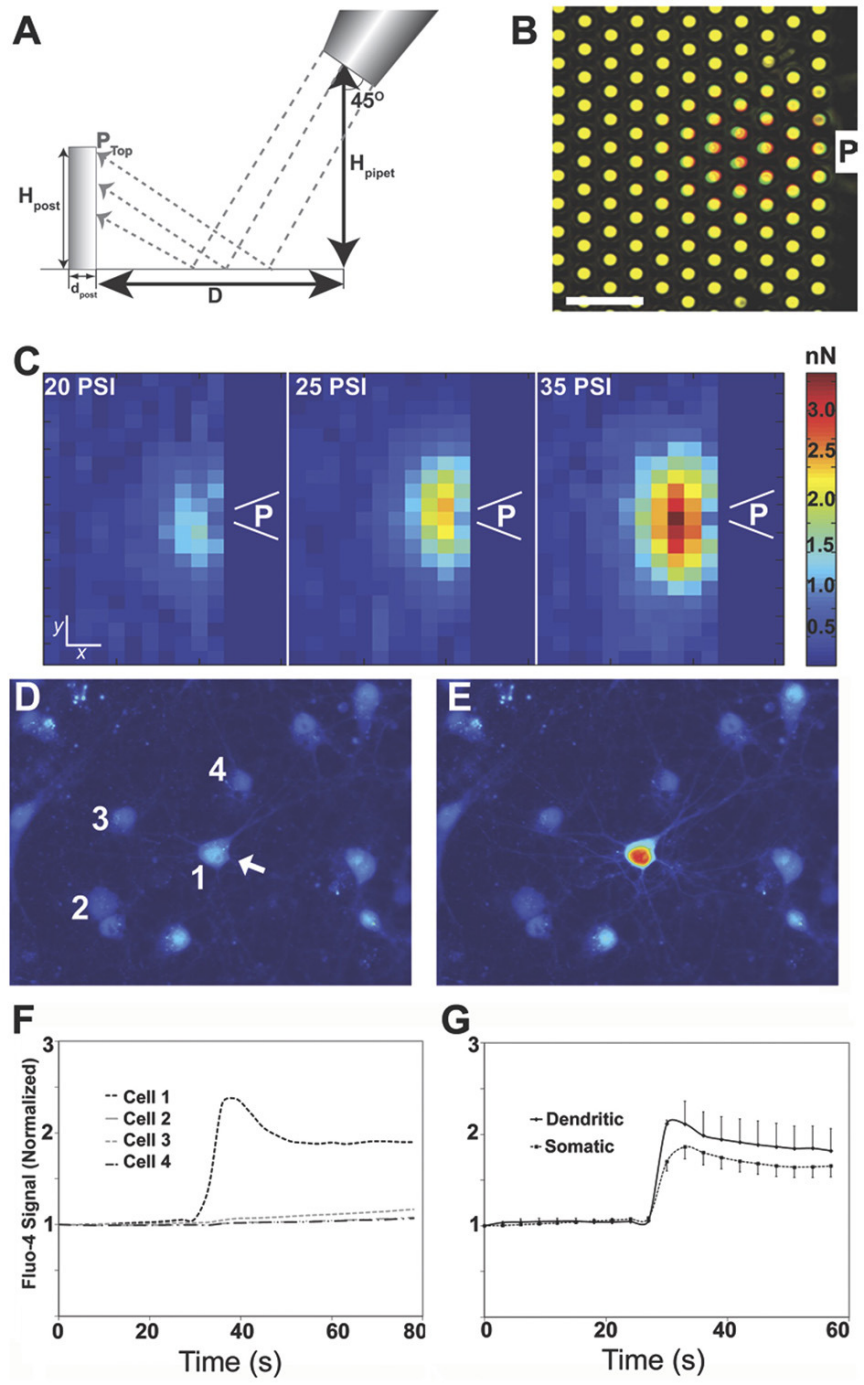
A system for injuring focal areas of a neuronal network. Micro-fabricated post array detectors (mPADs) were used to characterize the pulse field properties. The geometry of the system **(A)** could be varied by controlling the parameters of pipette height, Hpipet, and distance **D**. The fabricated posts were cylinders [8.3 μm height (Hpost), 1.83 μm diameter (dpost) arranged in a close-packed hexagonal array with a 4 μm post to post spacing]. **(B)** Overlay image of top of posts before (red) and during (green) pulse application (scale bar = 10 μm). **(C)** Computed force field for a 5 μm pipette placed at a 45° angle 10 μm above mPADs with an input pressures of 20, 25, 35 PSI. **P** indicates the location of the pipette. Scale bar = 3.5 and 4 μm for x and *y* coordinates, respectively. Pulse injuries could then be calibrated to disrupt calcium homeostasis for a single neuron. **(D)** Baseline calcium values just prior to the injury of neuron #1, scale bar = 10 μm. **(E)** Significant calcium increase in the injured neuron. **(F)** Trace of intracellular calcium for neurons 1–4 over time. **(G)** Mean results from five independent experiments in which the force was directly applied to the soma and calcium became elevated throughout a single cell. In these studies, the soma visibly deformed; [Ca^2+^]i significantly rose within 1 s of force application (170 ± 29%, *p* < 0.001 in somas, including nuclear calcium, *n* = 9 and 212 ± 74% in dendrites, *p* < 0.05, *n* = 11).

A force calibration method using micro-array post detectors (mPADs) was implemented to measure the pressure field applied. Again, the micropipette was steered to a known distance and height at a 45° angle from the target, an elastomeric substrate designed with 8 μm tall posts. A minimum of five measurements were taken for each input pressure to measure the post position at maximal deflection. The geometry of the set-up is illustrated in Figure 1A. The 45° angle of the micropipette resulted deflection across several posts, with the largest deflection in areas closest to the input pressure source. The overlay image of before (red) and during (green) mPAD deflection in Figure 1B illustrates a typical deformation field generated by a 5 μm pipette under a system input pressure of 20 PSI (scale bar = 10 μm). Figure 1C shows force field data for input pressures of 20, 25, and 35 PSI. It was determined that mPADs with an end load spring constant of 7.22 nN/μm were ideal for calibrating the forces needed to deform neurons. The range of possible fluid pressure pulse conditions corresponds to a force on the range of 0.4–1.2 nN applied to the post with a 20 PSI system input and a 5 μm micro-pipette (Supplementary Information 1). Similarly, pressure estimates are estimated to be ~0.004–0.04 PSI for these conditions. To confirm that the pulse injury results in local mechano-chemical activation, Fluo-4 imaging was performed to measure relative intracellular calcium levels in cultured cortical neurons. Micropipettes of varying sizes were tested, but those with ~5 μm bore diameters were ideal because they generated enough pressure to stimulate the neuron but still had a pulse field that spanned an area no greater than a single soma. Not all cells responded to the same pulse pressure, but the typical effective output force was estimated to be in the 0.5–4 nN range (0.004–0.04 PSI) from the mPAD data. Mechanically stimulating the somas of neurons resulted in a rapid increase in [Ca^2+^]_i_ from baseline, within one second of force application, which remained elevated for the remainder of the 1 min imaging sequence (Figures 1D–G). Across several individual somas from separate cell culture networks, we observed that [Ca^2+^]_i_ significantly rose within 1 s of force application (170 ± 29%, *p* < 0.001 in somas, including nuclear calcium, *n* = 9 and 212 ± 74% in dendrites, *p* < 0.05, *n* = 11). In cases where the mechanical force was sufficient to induce immediate plasma membrane damage resulting in CBXR dye entry, the Fluo-4 or GCaMP fluorescence rapidly declined to zero within seconds; this was assumed to be due to dilution of the cytosolic levels of Fluo-4 or GCaMP with entering extracellular fluid.

### Calcium Imaging and Injury Protocol

For calcium imaging experiments lasting <30 min, culture medium was exchanged with 2 ml extracellular saline (140 mM NaCl, 5 mM KCl, 2 mM CaCl_2_, 0.8 mM MgSO_4_, 10 mM HEPES, and 25 mM D-glucose, Sigma-Aldrich) at pH 7.4. The intracellular calcium indicator Fluo-4-AM was solubilized with the non-ionic surfactant Pluronic-F127 in DMSO. The dye solution was diluted into the extracellular saline solution for a final concentration of 1–5 μM. Cultures were loaded with the Fluo-4-AM solution for 30 or 45 min. Cultures were rinsed gently three times before imaging and then maintained in warm extracellular saline on a dish heater (Warner Instruments, Hamden, CT) during the imaging sequence. For calcium imaging studies spanning several hours, neurons were transduced for 7 days with adeno-associated virus expressing a genetically encoded calcium indicator, GCaMP3, GCaMP5G, or GCaMP6f under control of the synapsin-1 promoter (Tian et al., 2009) (GECI of Janelia Farm and Penn Vector Core #P1627, #P2478). Cultures were rinsed gently before imaging and then maintained in extracellular saline supplemented with amino acids (0.4 mM Glutamax™, 10 μM glycine, and MEM standard amino acids (Invitrogen #11130) on a dish heater or within a 37°C incubation chamber during the imaging sequence. For time periods in between imaging sequences, cells were maintained in MEM-based medium (#51200 Invitrogen) with supplements (0.4 mM Glutamax™, 10 μM glycine, D-glucose to 25 mM) and returned to an incubator at 37°C and 5% CO_2_.

A calibrated micropipette was filled with extracellular saline and steered to a measured distance and height away from the cell, dendrites, or axonal tracts of interest. Force was applied as a 100 ms air pulse driven and controlled by the FemtoJet device set at a known input pressure (0–40 PSI). The air pulse forced saline fluid from the micropipette and onto the target of interest. In some imaging sequences, the micropipette was then steered to subsequent targets for multiple fluid pulse injuries. Before, during and after injuries, images were stream-acquisitioned at 10 Hz with a Nikon Eclipse TE2000 microscope fitted with a spinning disk system (CSU-10b, Solamere Technologies), a CCD camera (Photometric Cool-Snap HQ2, BioVision, Exton, PA), a 488-nm excitation laser (Bruker Nano, Middleton, WI), and a Nikon 10x Plan Apo objective (N.A. = 0.4).

### Image Analysis

Individual cells were segmented as regions of interest using algorithms within MetaMorph 7.7.4 or MetaFluor 7.6.5 analysis software (Molecular Devices, Downington, PA) and by hand-selection methods implemented with ImageJ (NIH, Bethesda, MD) or Photoshop (Adobe System, San Jose, CA) software. Custom MATLAB scripts were used to process image stacks for somatic fluorescence values over time and then to detect calcium peaks of individual cells using a continuous wavelet transform method or through correlation analysis with a library of calcium transient waveforms (Patel et al., 2015). From the temporal information of detected calcium peaks, the phase synchronization matrix of pair-wise neurons, a global synchronization index (SI) ranging from 0 (uncoordinated activity) to 1 (fully synchronized activity), and the participation index (PI) of individual neurons within the network were computed using previously published eigenvector-based techniques (Li et al., 2007; Patel et al., 2012). In brief, each neuron *j* has a sequence of spike times denoted as *t*_*j*_*(n)* and the instantaneous phase of each neuron was computed for the nth inter-spike interval as:

$$\begin{array}{l} \varphi \left(t,n\right)=2\pi n+2\pi \frac{t-t_{j}\left(n\right)}{t_{j}\left(n+1\right)-t_{j}\left(n\right)} \end{array}$$

A phase synchronization matrix was then computed as the circular variance of the phase difference for pairwise neurons j and k:

$$\begin{array}{l} S_{jk}=|e^{i\left(\varphi_{j}\left(t\right)-\varphi_{k}\left(t\right)\right)}| \end{array}$$

Surrogate time series data were generated using an amplitude-adjusted Fourier transform of the original data and a phase synchronization matrix was constructed for the surrogate data. Eigenvalues were computed for both the original and surrogate phase synchronization matrices and denoted as λ_*k*_ and λ'_*k*_, respectively, with k = 1,…,M. The synchronization index was then calculated using the following formula:

$$Syn\_Index_{k}=\{\begin{array}{ll} \left(\lambda_{k}-{\bar{\lambda }}_{k}{}^{\prime })/(M-{\bar{\lambda }}_{k}{}^{\prime }\right) & if\;\times \lambda_{k}>({{\bar{\lambda }}^{\prime }}_{k}+2SD_{k})\\ \;0, & otherwise \end{array}$$

as developed by Li et al. (2007), where SD is the standard deviation of λ'_*k*_ and M is the number of neurons. The global synchronization index is the maximal value of the synchronization indices. The participation index (PI) is calculated by combining eigenvalues and eigenvectors of the synchronization matrix to indicate the contribution of each neuron to clusters of synchronization. In general, the closer the PI value was to 1.0, the more common the neuron showed a transient at the same time as other transients within the entire network. Functional connectivity index (CI) was statistically determined as a significant interaction between the spike patterns of two neurons in comparison to the surrogate time series and reported as the number of functional connections per neuron normalized by the total number of neurons within a field of view. A CI value of 1 indicates that the neuron is connected to every other neuron in the field of view, while a CI value of 0 indicates the neuron is not connected to any of the neurons. Finally, the network topology was determined by computing the overall modularity on a scale of 0–1, where 0 indicates a fully connected community structure and 1 indicates the presence of non-overlapping modules (Newman, 2006).

### Computational Model of Network Dynamics

To predict changes in structure and calcium dynamics that would occur if we randomly injured neurons in a neuronal network, we adapted the stochastic integrate-and-fire model previously developed in our lab (Patel et al., 2012), which incorporates AMPA and NMDA currents computed from previous Smoldyn simulations that give a temporal profile of the number of open receptors: N_AMPA_ and N_NMDA_ (Singh et al., 2011).

The network was built from 60 neuronal nodes with simulated axons, dendrites and synapses. These nodes were placed within a simulated square field of 10,000 μm^2^; by extending their axons in a random direction, we connected the nodes. From the axon, a Poisson distribution function with an expected value of 20 neighboring nodes was used to make connections to other nodes with weighted random sampling, which weighted closer neurons more heavily. Each neuron was then given dendrites using a cumulative distribution function based upon the average and standard deviation of the number of dendrites found *in vitro*. The inputs from connected neurons were assigned to specific dendrites through synapses. Each neuron was assigned either an excitatory or inhibitory phenotype, resulting in a 1:5 ratio of inhibitory to excitatory neurons; in turn, the excitatory synapses were populated with AMPA and NMDA receptors, whereas, the inhibitory neurons were given GABA receptors. On average, each synapse possessed 80 AMPA receptors and 20 NMDA receptors, or 12 GABA receptors.

As previously reported, ordinary differential equations (ODE) modeled the temporal profile for the state of the individual receptors and for stochasticity, the ODE results were augmented to include a stochastic element generated in Smoldyn (Singh et al., 2011). With the knowledge of receptor state, we computed the change in membrane potential by utilizing a leaky integrate-and-fire algorithm.

For each neuron, the change in membrane voltage (V_m_) is related to a sum of currents (I_i_) through membrane capacitance (C):

$$\begin{array}{l} C\frac{dV_{m}}{dt}=\underset{i}{\sum }I_{i} \end{array}$$

The total current includes sources from a leak factor, I_rest_, to account for the natural permeability of the membrane to ions, a current for membrane hyperpolarization from the calcium-potassium exchangers (I_KCa_), a current to model a spiking input followed by a refractory period (I_spike_) of regular spiking neurons (RS), a current to model low-threshold T-type calcium channels (I_LT_) of intrinsic bursting (IB) neurons, and finally a synaptic current from AMPA and NMDA receptors, or loss from GABA receptors (I_syn_). Together, the currents are as follows:

$$\begin{array}{l} \underset{i}{\sum }I_{i}=I_{rest}+\;I_{KCa}+I_{LT}\;{+\;I}_{spike}+I_{syn} \end{array}$$

Current flow is modulated by ionic conductance g_r_ of each receptor:

$$\begin{array}{l} I_{r}=-g_{r}\left(V_{m}-E_{r}\right)\;x\;N_{r} \end{array}$$

where E_r_ is the reversal potential for a given receptor type and the number of open receptors (N_r_) scales the current value to represent current from all open receptors. It was assumed that gAMPA, gNMDA, and gGABA, the single channel conductance for each receptor, was 12, 45, 40 pS, respectively. The current and membrane voltage for each neuron was computed for every 2 ms time-step over a 60 s time period.

The number of available NMDA receptors was also affected by their probability of containing a magnesium block. The probability of a magnesium block on the NMDA receptors was dependent upon membrane voltage and magnesium concentration and computed as:

$$\begin{array}{l} P_{unblocked}=\frac{1}{1+e^{-0.062V_{m}}\cdot \frac{\left[Mg^{+2}\right]}{3.57}} \end{array}$$

Finally, the number of calcium ions entering the neuron were modeled as entering through NMDA channels with a conductance g_NMDA_:

$$\begin{array}{l} N_{Ca++}=\frac{g_{NMDA}\cdot V_{m}\cdot N_{NMDA}}{Z_{Ca++}\cdot e_{C}} \end{array}$$

The valence for calcium is Z_Ca_ and the elementary charge is e_c_ Partial buffering of the calcium occurs at the spines, and the remaining free calcium is summed for each cell.

To simulate an injury, a subset of neurons were chosen to be injured such that simulations were done for 0, 13, 25, 50, and 75% of the cells in the network. A rise in intracellular calcium to a concentration of 2 mM (extracellular calcium concentration) held constant for 60 s simulated in injury in which disturbed calcium homeostasis prevents the neuron from firing. The impact of “injured” neurons on the activity of the “uninjured” neurons was then computed.

### Viability Analysis

At 6 h post-injury, cell survival was determined with a fluorescence-based assay. Cells were incubated for 30 min with both Hoechst 33342 (Invitrogen) and membrane-impermeable ethidium homodimer (EtHD, Sigma-Aldrich) to stain total and dead/dying cells. Fluorescent images were taken at three wavelengths with FITC, TRITC, and DAPI filter sets to detect GCaMP, EtHD, and Hoechst, respectively. Tri-color images were then registered to GCaMP images taken previously in the injury sequence to correlate the injury magnitude of individual cells with their viability status at 6 h post-injury.

### Poration Assay

To determine which cells underwent membrane poration, 5(6)-carboxy-X-rhodamine (CBXR, Santa Cruz #sc-210421), a membrane impermeant dye, was added to the extracellular solution at 20 μM and imaged with 561-nm excitation laser both before and after the pulse injury application. During the injury, 488-nm excitation was utilized to track the intracellular calcium dynamics. Pre-images were digitally subtracted from post-images to detect an intracellular increase in CBXR for each cell.

### Mitochondrial Membrane Potential Assay

Changes in the mitochondrial membrane potential (ΔΨ_m_) were estimated with the indicator rhodamine 123 (rh123). When mitochondrial membranes are polarized, the dye accumulates in the membrane and its fluorescence quenches. With calcium entry and depolarization of the mitochondria, the dye redistributes to the cytoplasm, where rh123 fluorescence intensity increases. Cultures were loaded with 10 μg/ml rh123 for 10 min and washed three times with extracellular saline plus amino acids. Z-stacks were taken with low-power 488-excitation before injury for a baseline measurement. During injury or sham, images were stream-acquisitioned at 10 Hz for 2 min to monitor the immediate effect of the injury on ΔΨ_m_. After injury, stacks were taken immediately and again at 15 min post-injury. To obtain the maximal rh123 signal, mitochondria were depolarized with a 2 min treatment with the protonophoric uncoupler FCCP (Carbonyl cyanide 4-(trifluoromethoxy)phenylhydrazone) at 0.2 μM, and a final z-stack was taken. Maximum projections of the z-stacks were computed, images were segmented into individual cellular regions, and mean intensity values were computed for each.

### Statistics

Statistical differences in intracellular calcium values (reported as ΔF/F_o_, F_max_/F_o_, or ∫ Fdt) and network properties (calcium event rate, synchronization and participation) were determined using *t*-tests, ANCOVA, two-way ANOVA, or one-way ANOVA and *post-hoc* Tukey's when appropriate (MS Excel and JMP Pro). To determine which factors cell death depended upon, logistic regression analysis with Chi-squared tests and Fisher's linear discriminate analysis with *F*-test (Wilk's lambda) were employed. Significance was met at an alpha level of 0.05.

## Results

**Figure 2:**
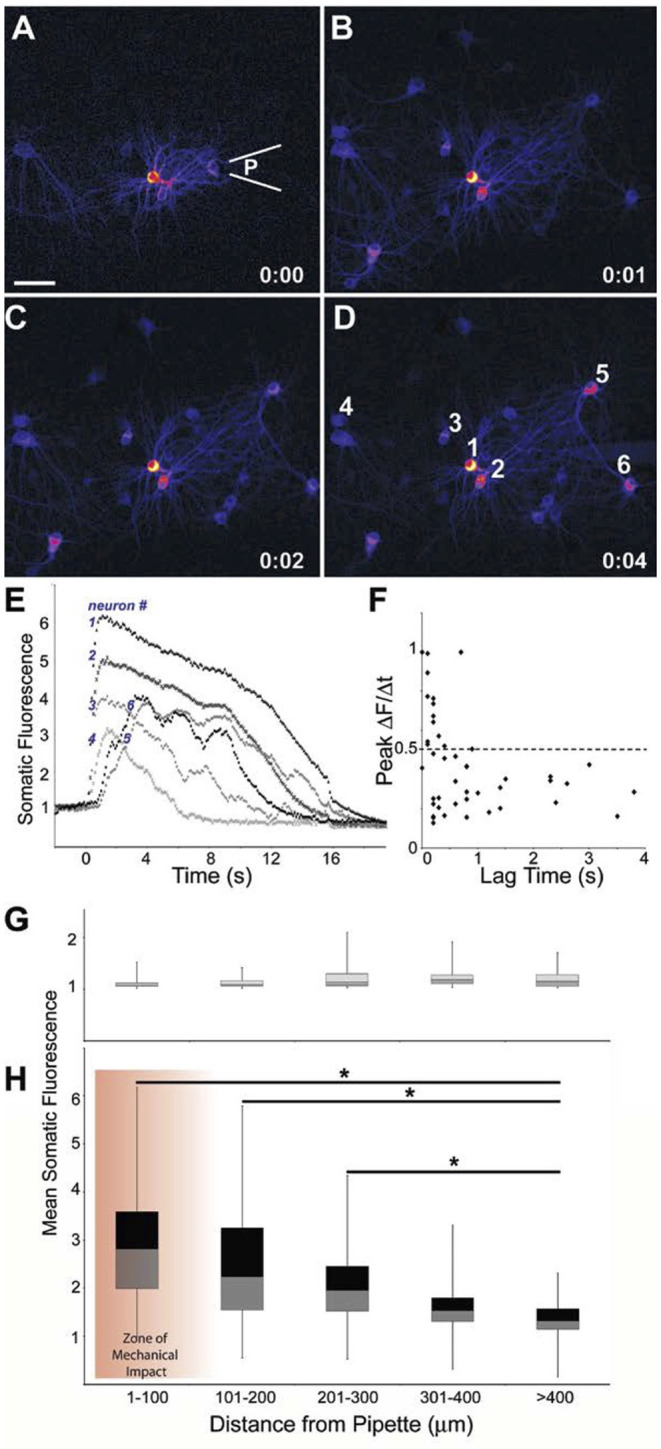
Primary responses within and outside the zone of mechanical impact. Difference images indicating change in Fluo-4 fluorescence from baseline to **(A)** immediately after (100 ms) injury by a pulse from a pipette a location (**P**), **(B)** 1 s post- injury, **(C)** 2 s post-injury, **(D)** 4 s post-injury. Scale bar = 40 μm. **(E)** Traces for Fluo-4 intensity of the 6 indicated neurons. Neurons 5 and 6, positioned upstream of the injury pulse, display a latent response. **(F)** A more latent response to injury yielded a slower rise in intracellular calcium (least squares regression analysis: *p* < 0.01, *n* = 46 neurons). Mean somatic fluorescence values vs. distance from the pipette for **(G)** baseline conditions and **(H)** peak-injured conditions showed that the injury response tapered significantly with distance but existed outside of the “zone of mechanical impact” (*p < 0.01).

On the timescale of seconds, we observed three characteristic responses to targeted micromechanical stimulation. The majority of the responses classified as either (1) primary or (2) secondary responses occurring over the course of seconds (Figures 2A–D). In a third and less common case (<5% of the neurons within the field of view), the neurons showed a rapid decrease in Fluo-4 or GCaMP fluorescence. Subsequent experiments showed that neurons in this third category demonstrated an immediate increase in membrane permeability (“mechanoporation”) from this local mechanical force. In comparison, neurons subjected to injuries without poration showed a transient increase in fluorescence that gradually returned to pre-stimulation baseline values (Figure 2E). For these transient events, the increase in fluorescence was either immediate, reaching a peak value in <0.5 s, or as in the case of a secondary injury, the increase in Fluo-4 or GCaMP levels was delayed, achieving a peak value with a latency of >0.5 s to several seconds (Figure 2F). Longer term studies tested whether the mechanical injuries led to cell death at 6 h post-injury. Overall, the outcome of cell death was significantly dependent upon the peak change in [Ca^2+^]_i_ (logistic regression analysis, *p* < 0.01, *n* = 170 cells from *N* = 5 independent trials).

The primary injuries predominantly occurred within a 100 μm zone of the mechanical stimulation (see section Methods). In comparison, the delayed increase in fluorescence would appear both within the field of injured neurons and outside of the zone of mechanical impact, resulting in a mean increase in fluorescence that progressively declined with distance from the central location of applied physical force. As indicated by Fluo-4 fluorescence, a mean somatic peak of 291 ± 124% above baseline was reached by the cells within 100 μm of the injury site, cells at a distances >400 μm from the pulse injury, and had a significantly lower mean somatic fluorescence of 143 ± 37%, *p* < 0.001, single factor ANOVA) (Figures 2G,H). Furthermore, mean changes in somatic fluorescence were not significantly different for areas within 1–100 and 100–200 μm of the stimulation point. We, therefore, grouped neurons within these two areas as a single group and termed this area as the “injured region” for a subset of analyses. Likewise, we grouped neurons beyond 200 μm of the stimulation area in a second group and termed this area as the “penumbra region.”

**Figure 3:**
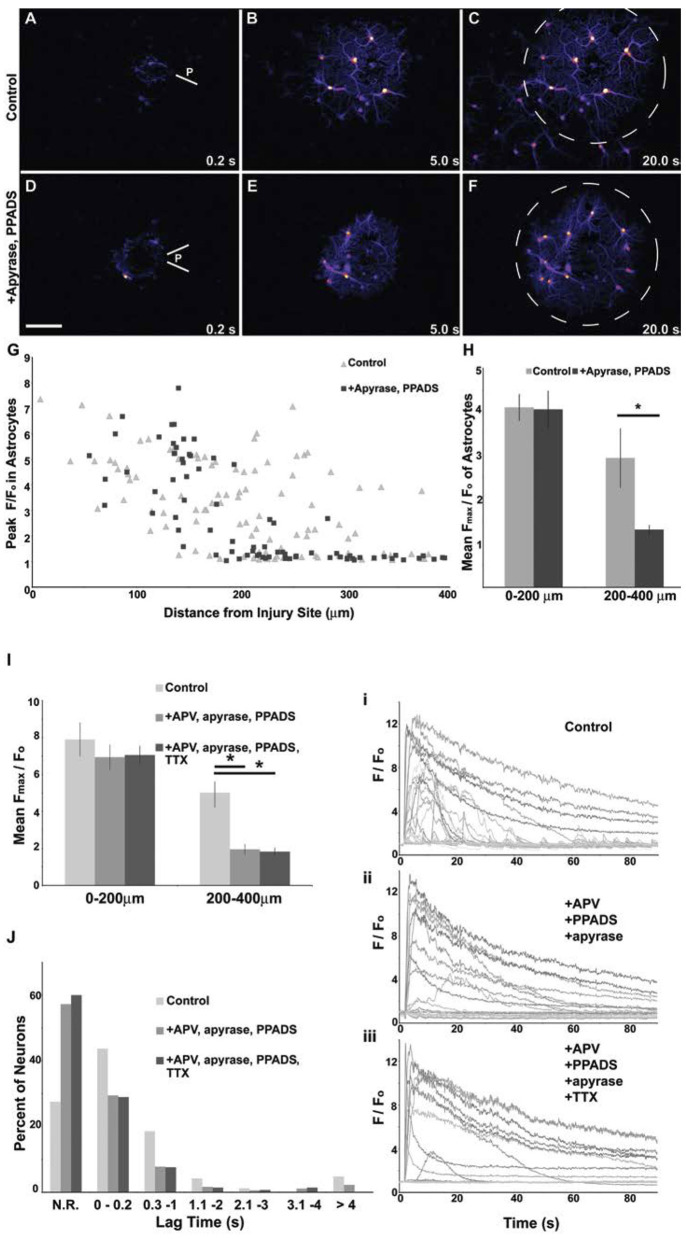
Attenuation of the primary mechanical response in areas distant from mechanical injury. Difference images showing changes in Fluo-4 fluorescence in a culture containing a network of astrocytes mixed with neurons. With a 200 ms mild pulse injury **(A,D)**, astrocytes at the injury site (**P**) had a rise in intracellular calcium, scale bar = 100 μm. Subsequently, a wave of increased intracellular calcium spread circumferentially from the injury site through the astrocyte network as shown by images at 5 s **(B,E)** and 20 s **(C,F)**. The application of 20 U/ml apyrase and 10 μm PPADS prevented the wave from spreading beyond a 200 μm radius (dashed line circle). Peak fluorescent values were attenuated in more distal astrocytes with drug application **(G)**. The population mean was computed for peak fluorescent values of astrocytes and was not affected by drug treatment within 200 μm of the injury, but was attenuated by drug treatment in astrocytes 200–400 μm from the injury site **(H)**, *p < 0.05. Likewise, injury propagation to more distal neurons can be blocked with a combinatorial drug treatment target ATP and NMDA signaling. The population mean was computed for peak fluorescent values of neurons and was not affected by a combination of 50 μm APV, 20 U/ml apyrase and 10 μm PPADS treatment within 200 μm of the injury but was attenuated by drug treatment in neurons 200–400 μm from the injury site **(I)**, *p* < 0.01. The application on 1 μm TTX blocked activity but provided no further decrease in peak [Ca^2+^]i post-injury. The lag time from injury application to the onset of the rise in intracellular calcium was measured for all neurons. Neurons that did not reach a 2-fold increase in [Ca^2+^]i were categorized as “no response” (**N.R**.). A greater percentage of neurons responded to the injury under control conditions than in drug treatments **(J)**. Secondary responses (lag time >0.2 s) were less frequent in drug treated neurons. Significant changes in [Ca^2+^]i at time points >4 s post-injury were eliminated with TTX treatment. Example traces for control (i) and drug treated (ii, iii) injury responses are shown.

A single micromechanical pulse consistently triggered a localized increase in [Ca^+2^]_i_ in the target neuron, radially outward over time to activate neighboring neurons (Figures 3A–C). Purinergic signaling, and in particular ATP receptor signaling, is one primary mechanism for transmitting force-triggered calcium waves through astrocyte networks (Neary et al., 2003; Choo et al., 2013). One consideration was whether the micromechanical pulse elicited a response in the astrocyte networks within the mixed culture, leading to a progressive activation of neuronal networks distant from the site of initial injury. To this end, the application of both apyrase, which catalyzes the hydrolysis of ATP, and PPADS, a P2X purinergic receptor antagonist, significantly attenuated the wave beyond a 200 μm radius from the injury site as indicated by difference images (Figures 3D–H) and the mean F_max_/F_o_ value (2.95 ± 0.68 for control vs. 1.31 ± 0.11 for treated, *p* < 0.05). This treatment also led to a significant reduction in neuronal cytosolic calcium changes in regions remote from the site of localized physical injury (Figures 3I,J). Within the injury zone, we used a combination of NMDAR blockade with APV to account for the possible activation of mechanosensitive NMDARs and the application of tetrodotoxin to examine if key parts of neurotransmission contribute to the primary injury response within the injury zone. However, the combination of treatments to block purinergic signaling and activity-induced changes in cytosolic calcium did not significantly change the immediate (<0.5 s) changes in intracellular calcium for neurons within 200 μm of the injured neuron (Figure 3I). Together, these data suggest that the response to a focused mechanical injury to a mixed neuronal and glial population can be divided into two physically separate areas—the injured region and the region distant from the region. The mechanism regulating the response of the neurons in these two areas is distinct: purinergic signaling through astrocytes is key for extending the effects of targeted mechanical stimulation to remote (>200 μm) network regions, while it is not a primary regulator of the response within 200 μm of stimulation.

**Figure 4:**
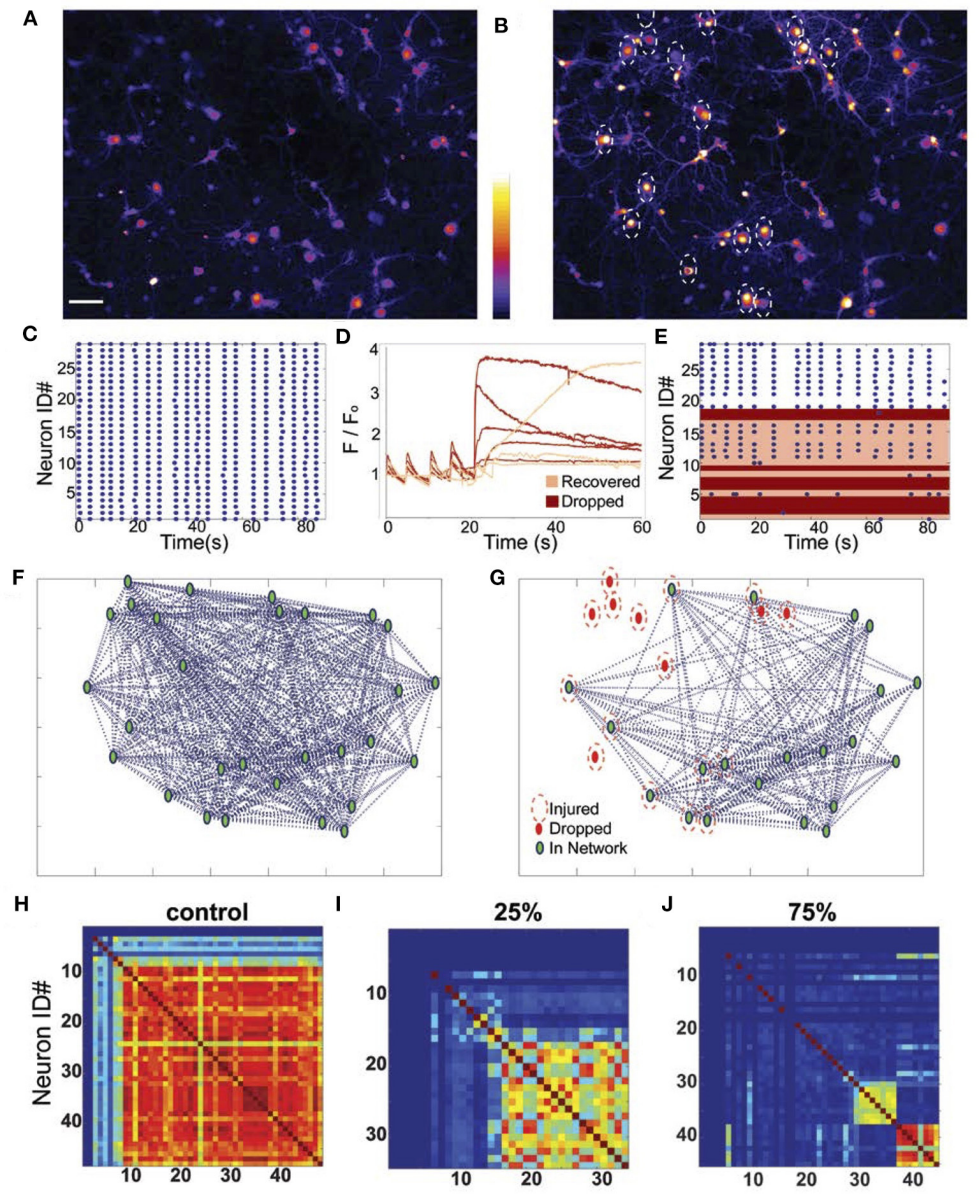
Representative changes in network structure and recovery after micromechanical injury. Calcium levels of neural networks at baseline **(A)** and post-injury of 60% of the neurons (indicated by white dashed circles; astrocytes also displayed increased calcium with injury but were not included in the network analysis). **(B)** At baseline, the neural networks displayed synchronous activity as indicated **(C)** visually by their raster plots. **(D)** Plot of normalized fluorescent levels over time of several neurons that were injured at the 20 s time point. When the intracellular calcium rose rapidly and immediately with force application, the neurons dropped out of the network (red traces), but when the injury induced a slow or moderate rise in calcium, neurons rapidly recovered and reintegrated within minutes (pink traces). **(E)** Raster plots of neuronal activity showing the recovery (pink) and loss (red) of injured neurons 2 min after injury. Network topology typically showed a fully connected network under baseline conditions (F; green marker = connected neuron, pink trace = injured neuron, red marker = disconnected neuron), but functional, network topology immediately changed as neurons dropped out of the network with injury **(G)**. Synchronicity matrices **(H–J)** show a progressive decline in cell-to-cell synchronization with increasing levels of injury.

We next examined if these focused mechanical stimulations produce longer lasting changes to the network structure and neuronal activity patterns. In parallel, we developed an *in silico* model to predict the changes in structure and calcium dynamics that would occur if we randomly “injured” neurons in a neuronal network of a size similar to the *in vitro* network. In the experimental studies, we varied the injury zone size by targeting multiple points within the field of view, effectively injuring 0–100% of the neurons within a 455 μm field. For the simulations, injury was modeled as a sustained (60 s) elevation in [Ca^+2^]_i_ for a fraction of excitatory and inhibitory neurons from the network of 60 neurons. Using analysis methods to infer the synchronization and functional connections among neurons, we observed that mature control neural networks (DIV 18–22) consistently displayed oscillatory and synchronized activity in calcium transients (average rate of 0.35 ± 0.11 Hz; typical transient activity pattern in Figures 4A,C). Prior to mechanical injury, the mean, global synchronization index (SI_pre_) of networks was 0.59 ± 0.12 (*n* = 34 fields of view). Following injury to either single or multiple cell clusters within a 455 μm field of view (Figures 4B,D,E), the network topology typically evolved from a highly connected network (Figure 4F) to a reduced one as injured neurons dropped from the network (Figure 4G) on an acute time scale (representative changes in synchrony shown in Figures 4H–J).

**Figure 5:**
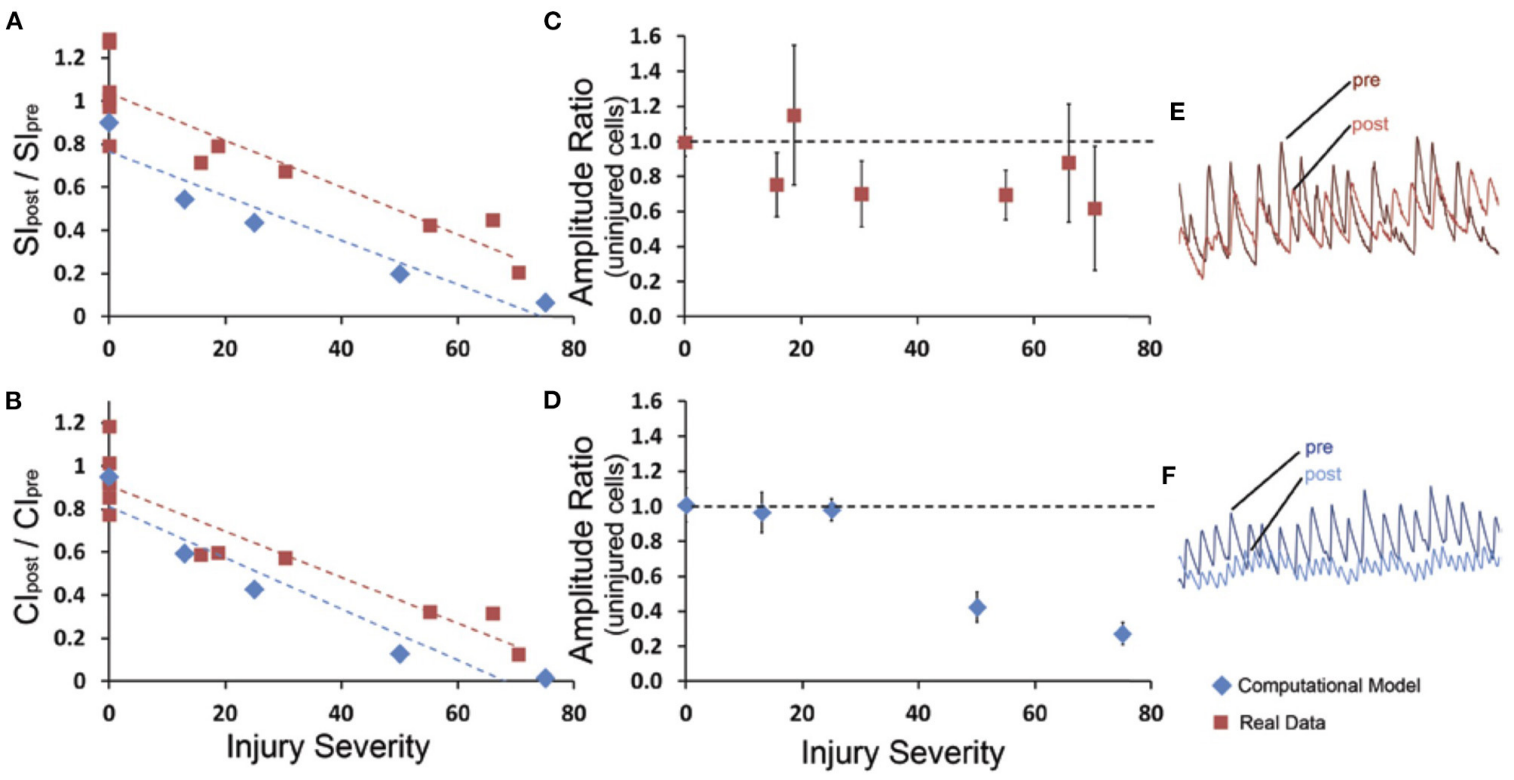
Network synchrony and connectivity declines with extent of micromechanical injury *in vitro* and *in silico*. **(A)** The global synchronization index ratio from post-injury to pre-injury was measured for at least five levels of injury in the computational model (blue) and in experimental data (red); there was a linear decline in the global synchronization index ratio (SIpost/SIpre). **(B)** Similar results were observed in the global connectivity index ratio (CIpost/CIpre). The rate of decline over injury levels for connectivity index and synchronization index was not different between *in vitro* results and *in silico* predictions. For the experimental data and the computational model **(C,D)**, a mean amplitude ratio (Apost/Apre) was calculated for the uninjured cells within the injured network. For the experimental data, the uninjured cells typically demonstrated a moderate decline in amplitude that was independent of injury level, whereas the computational model yielded a more severe drop in the amplitude that was injury severity-dependent. Examples of pre- and post- amplitude traces for experimental and computational **(E,F)** data.

In the field of view containing injured neurons, the ratio of the post- to pre-injury synchronization indices (SI_post_/SI_pre_) linearly decreased with increasing injury severity (*R*^2^ = 0.814, *p* < 0.001; Figure 5A). A similar effect was observed in the relative change in connectivity index (CI_post_/CI_pre_) measure immediately after injury (*R*^2^ = 0.859, *p* < 0.001; Figure 5B). Injury of the neurons in the *in silico* network led to a predicted decline in connectivity and synchrony that was not significantly different from the measured changes *in vitro* (Figures 5A,C). Interestingly, these changes in connectivity led to a moderate reduction in the peak fluctuations in [Ca+2]i for neurons that remained active within the network after injury *in vitro*, independent of injury level (Figures 5C,E). In comparison, injuring more than 30% of the neurons in the *in silico* network led to a sharp and significant decline in the amplitude of oscillations for active neurons in the network (Figures 5D,E). This difference may be explained by the fact that for *in vitro* networks, innervation by additional neurons outside of the field of view supported the network; whereas the *in silico* networks were limited to the 60 neurons incorporated in the model. Taken together, our experimental data show that focused injury only affects network topology and activity within the injury zone and does not extend significantly into uninjured areas, which generally agrees with our *in silico* results.

**Figure 6:**
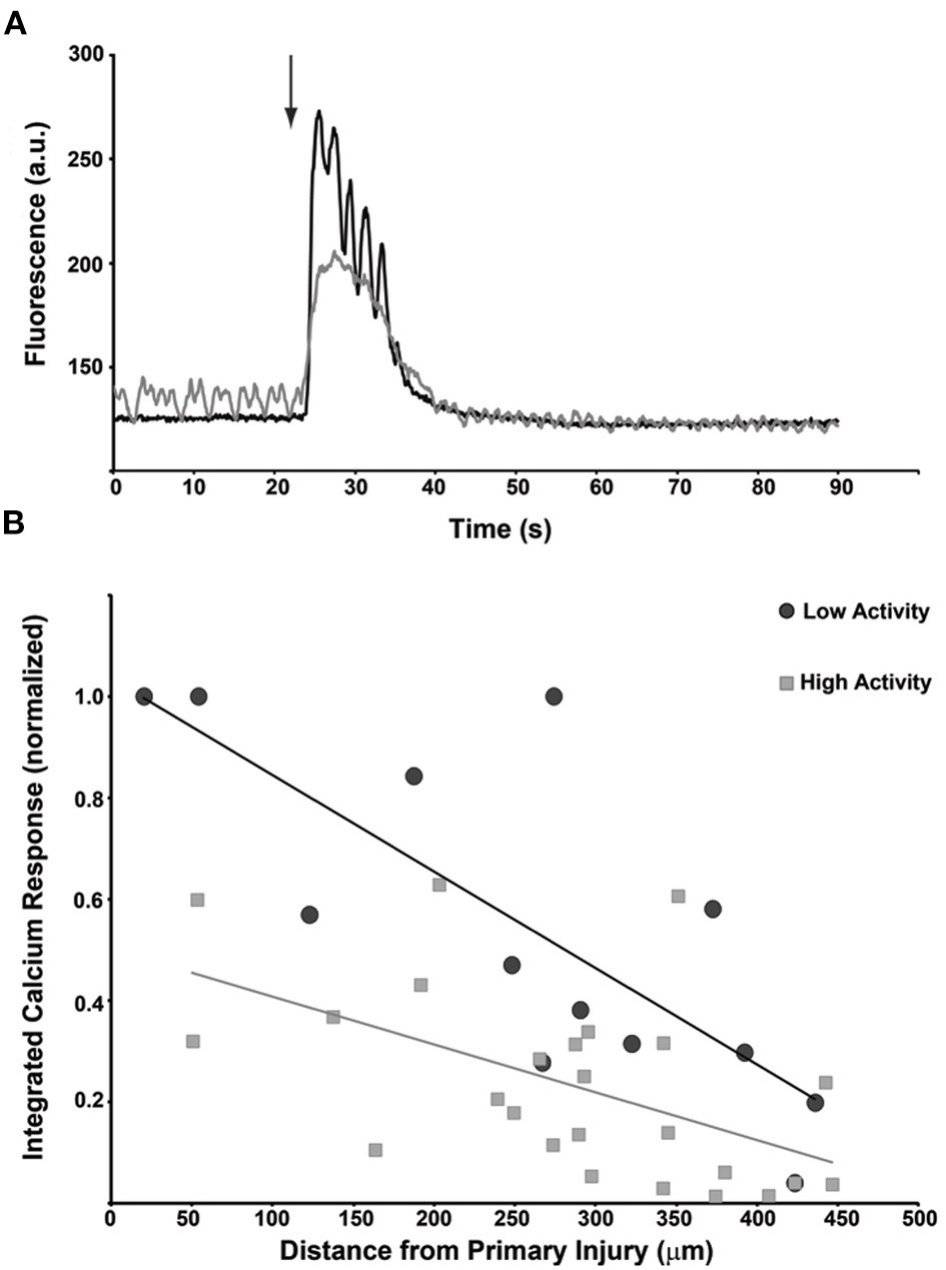
Primary injury response is dependent upon the activity state of individual neurons just prior to the injury. **(A)** Traces for two cells that are subjected to the same micromechanical impact at the same time. The induced calcium transient for the more electrically active cell (gray) reaches a lower maximum than the inactive cell (black). Interestingly, the activity of the quiescent cell briefly increases during the calcium transient. **(B)** Composite data from *n* = 40 neurons that were classified as having low activity (black circles) or high activity (gray squares). The transient calcium response to the injury was integrated over time and compared plotted against the distance from the injury epicenter. The induced calcium transient was dependent upon distance but was also greater in low-activity cells.

The relationship between network activity/structure and progressive injury demonstrates the clear role that microtrauma can play on the overall network capacity after trauma. However, it does not directly test if the activity level prior to microtrauma affects the subsequent activity and integration of neurons within the mechanical injury zone of a network. Across several independent networks, we classified neurons as either high activity, with distinct oscillations prior to injury, or low activity with no detectable [Ca^+2^]_i_ transients before injury (Figure 6A). We measured the resulting response to a single micromechanical stimulation by integrating the [Ca^+2^]_i_ transient over the first minute following injury. Neurons with low activity showed a much more robust response to micromechanical stimulation than neurons with high activity (Figure 6), demonstrating that the activity state of neurons is a significant contributor to the post-acute injury response.

At this point, we directed our attention to relating features of the initial injury response to the long-term outcome of neurons within the network. Although we had found that the outcome of cell death was significantly dependent upon the peak change in [Ca^2+^], this single factor is not a reliable predictor for cell fate because we also observed recovery from changes in [Ca^2+^]_i_ on the order of 600% of baseline in the absence of membrane tearing; we also found that uninjured neurons often responded to the micromechanical stimulation, and at 6 h, show propidium iodide labeling indicating cell death. To develop a more complete model of neuronal degeneration, we explored if network activity and topology are key factors in mediating neuronal survival both within the injured area and in the penumbra. Several past studies show that overactivation of a network or activation of specific subtypes of glutamate receptors within less active networks can lead to relatively rapid neuronal degeneration (Hardingham et al., 2002; Martel et al., 2009). Alternatively, modest synaptic stimulation can promote neuronal survival through production of antioxidants and stimulation of cell survival pathways (Léveillé et al., 2010).

**Figure 7:**
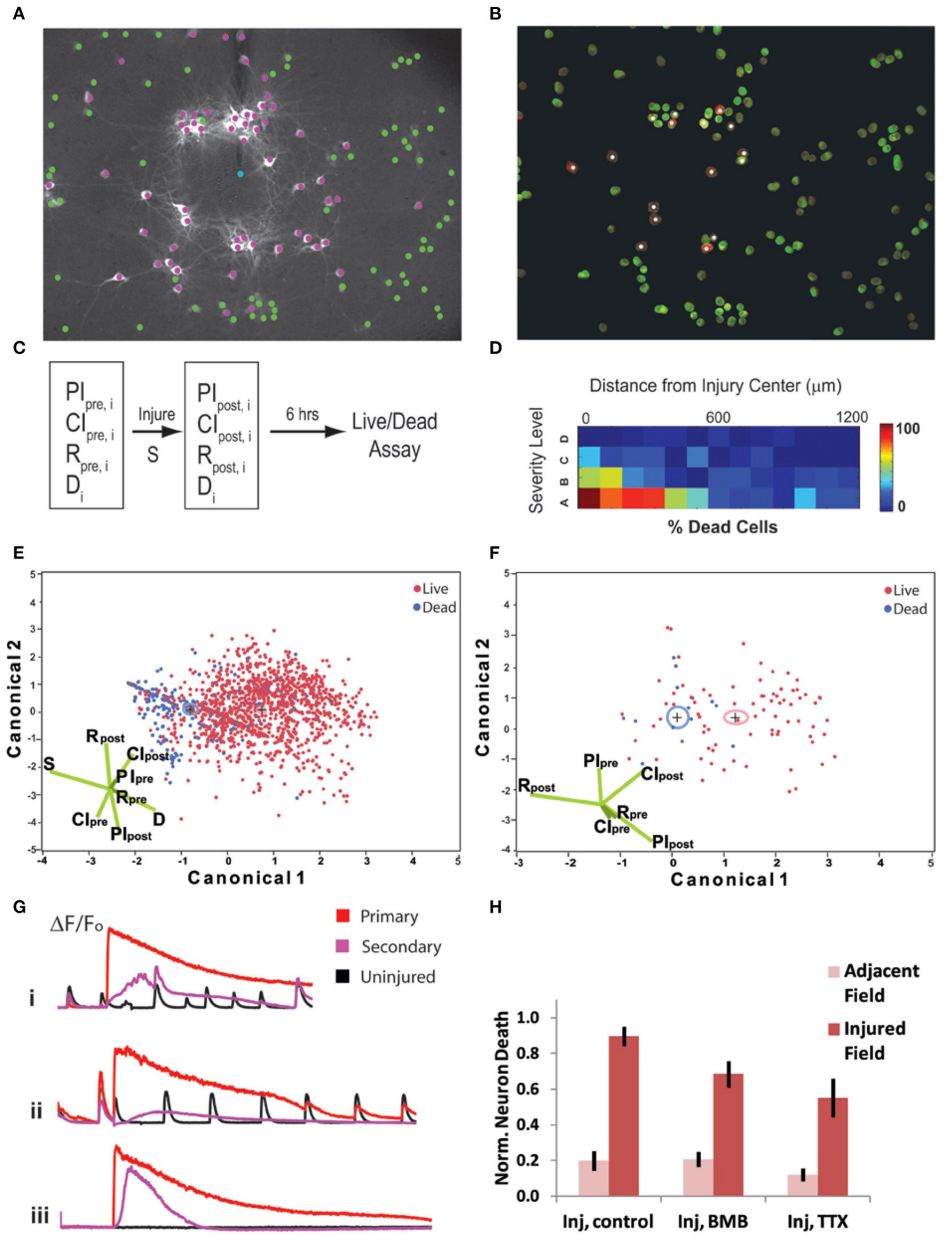
Cell fate is dependent on neuronal connectivity, injury severity, and primary injury response. Injury signature **(A)** when 50% of cells are injured (purple) and <5% undergo a poration injury (blue). **(B)** Cell death at 6 h, with white puncta/purple labeling indicating propidium iodide-positive cells. **(C)** Schematic of experimental procedure; eight parameters (CIpre, PIpre, Rpre, CIpost, PIpost, Rpost, S, and d) in total were measured for 1,399 individual cells from 14 independent trials (index on cells = n). **(D)** Gradient of cell death with respect to injury severity and distance from primary injury site. Cell fate is directly dependent upon injury severity and inversely dependent upon cell distance from the injury center (logistic regression analysis with Chi-squared test, *p* < 0.0001. **(E)** Cell fate was easily delineated by Fisher's linear discriminant analysis (79.8% correct prediction, Wilks' Lambda = 0.783) when using all six input parameters. **(F)** Reducing the predictive model to only the network parameters, CIpre, PIpre, Rpre, CIpost, PIpost, Rpost, results in a similar prediction for an individual group (distal, >500μm from injury site, with high severity) was still obtainable (77.3% correct prediction, Wilks' Lambda = 0.838). **(G)** Example traces of intracellular calcium dynamics for cells with a primary injury (red), secondary injury (purple), or no injury (black). Each trace is given for control (i), bicuculline (BMB) (ii) and tetrodotoxin (TTX) (iii) treatment conditions. **(H)** Neuronal death levels were normalized to control. Single factor ANOVA (*p* < 0.01) and post-Tukey (*p* < 0.01) analysis show that blocking electrical activity significantly decreases cell death.

We first categorized the severity (S) of injury using the percentage of cells with an elevated [Ca^2+^]_i_ >twice the baseline after mechanical impact (from highest severity to lowest: A = 75–100%, B = 50–75%, C = 25–50%, D = 0–25%) within the field of view (a 50–75% level injury is depicted in Figure 7A). We tracked cell death (Figure 7B) in cultures at 6 h post-injury or post-sham manipulation in the injured field of view and in an adjacent field for individual cells (n), and we registered these data in regards to several network properties that reflected the integration of individual neuronal activity within the network (participation index), the estimated density of connections in the network (connectivity index), and the rate of event activity within each neuron over time (R) (Figure 7C). As expected, the incidence of cell death at 6 h was directly dependent upon injury severity and inversely dependent upon cell distance from the injury center (Figure 7D; logistic regression analysis with Chi-squared test, *p* < 0.0001 for both severity and distance, *n* = 1,399 neurons from *N* = 14 trials). However, injury severity alone was a less powerful predictor of outcome when compared to an algorithm using all eight input variables for all cells (PI_pre_, CI_pre_, R_base_, PI_post_, CI_post_, R_post_, S, and d; 79.8% correct prediction from LDA, Wilks' Lambda = 0.783) (Figure 7E). We found that activity, functional connectivity, and participation alone predict cell fate (logistic regression analysis with Chi-squared test, *p* < 0.005). There was a significant relationship in which cell survival is dependent upon the activity rate and network participation measured after the injury (logistic regression analysis with likelihood ratio tests, R_post_: *p* < 0.001, CI_post_: *p* < 0.05); the participation and number of calcium peaks per minute had been greater in the surviving neurons in comparison to those that eventually died.

We extended our analysis linking activity-based measures with outcome into the distant penumbra (>500 μm from the injury site). We observed that only high injury levels led to a significant increase in cell death within this region, and thus, we focused our analysis in the penumbra region in this data subset. Similar to our analysis of the injured area, a set of 6 input variables (PI_pre_, CI_pre_, R_base_, PI_post_, CI_post_, R_post_) in a Fisher's linear discriminant analysis yielded significant separation of live from dead cells (77.3% correct prediction, Wilks' Lambda = 0.838) (Figure 7F).

Given that cell survival in both the penumbra and injury zone could be readily predicted by network participation and activity, we tested if either the active recruitment of neurons back into the network or activity blockade would affect neuronal fate. For this study, we kept the injury level constant so that 50 ± 10% of the cells would undergo an injury as indicated by a sustained rise in [Ca^2+^]_i_. We eliminated the small percentage (<5%) of neurons that underwent poration from the subsequent analysis, as previous data show that this level is not sufficient to alter activity significantly or influence neuronal degeneration of neighboring neurons. To modulate activity in the injured network, we either blocked GABA_A_ receptor signaling to promote network-wide bursting [50 μM Bicuculline Methbromide (BMB)] or we blocked activity completely [1 μM tetrodotoxin (TTX)] for a 6 h period post-injury. In all cases, the typical primary injury occurred (Figure 7G), however, with BMB, the cells more rapidly recruited back into the network, but with TTX, no activity was detected for all neurons. Remarkably, neuronal death was significantly reduced for the TTX-treated cultures (Figure 7H; one-way ANOVA, *p* < 0.01 and *post-hoc* Tukey, *p* < 0.01), and we found no significant reduction in death for BMB-treated networks. This result implies that network activity may be associated with factors that lend to survival, but active firing during and after the injury is not causally responsible for neuronal survival.

**Figure 8:**
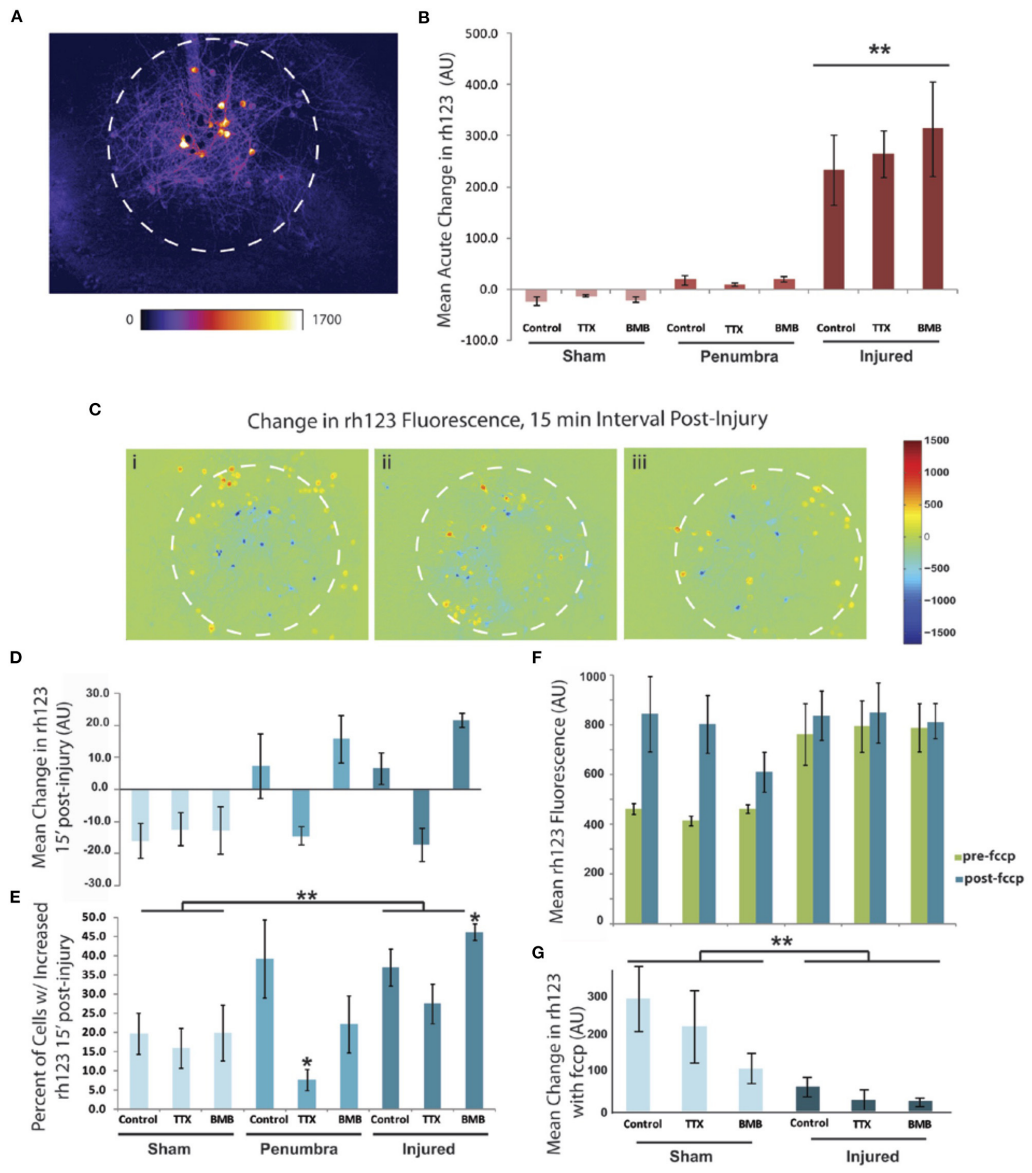
Injury-induced mitochondrial depolarization is exacerbated by increasing network activity but is reduced by blocking activity. **(A)** Fluorescent signal rh123, a mitochondrial membrane potential indicator, increases within the injury zone (approximated by the white dashed circles) immediately upon impact. Neurons outside of this zone do not have an acute response and were categorized as “penumbra.” **(B)** Quantitative data for the acute response of rh123 signal to the focal injury (two-way ANOVA, F = 28.1, *p* < 0.0001, with a significant injury effect, **p < 0.0001 for both injured vs. sham and injured vs. penumbra, *post-hoc* Tukey). **(C)** Difference images for changes in rh123 signal over the 15 min time period post injury (i: Control, ii: TTX, iii: BMB). **(D)** Quantitative data for the 15 min response of rh123 signal to the focal injury. Amplitude data were not significant but there was a trend for increased rh123 in the penumbra and injured region for both the control treatment and the BMB-treated cultures. All other conditions had a reduction in rh123 over this time period. **(E)** Data from D represented as percent of cells with an increased rh123 signal (two-way ANOVA, F = 5.51, *p* < 0.005, with both significant injury and drug effects: injured vs. sham, **p < 0.005 and TTX vs. control, *p < 0.05, *post-hoc* Tukey). Isolating the data in the injured category also revealed a significant increase in rh123 with BMB vs. control (*p < 0.05, *post-hoc* Tukey). **(F)** At the end of the study the response to 0.2 μM FCCP was measured. **(G)** The induced change was significant (two-way ANOVA, F = 5.39, *p* < 0.005 with a significant injury effect, **p < 0.005 for injured vs. sham, *post-hoc* Tukey). There was no significant drug effect, but BMB had a trending effect of compromising the FCCP response.

These data point to the neuroprotective effect of suppressing neural activity in the post-acute period after injury, but they do not provide direct insight into possible protection mechanisms. In other models of excitotoxicity and brain injury, excessive intracellular calcium ultimately leads to dysfunctional mitochondria (Ahmed et al., 2002; Abramov and Duchen, 2008). This disruption in bioenergetic homeostasis cannot meet the energetic demands of high synaptic activity and may lead to neuronal death (Harris et al., 2012). As such, we decided to test the impact of injury and varying synaptic activity on mitochondrial potential (ΔΨ_m_). We measured an immediate increase in rh123 fluorescence approximately within the 200 μm diameter of the focal injury (Figure 8A). We measured the rh123 fluorescence value at exactly 2 min after the injury on a cell-by-cell basis and binned the neurons into either group based upon this response at this time point to accurately subdivide the neurons into an “injured” and “penumbra” group. The acute increase was independent of network activity during the injury as there was no significant drug effect with BMB and TTX treatments (Figure 8B) (two-way ANOVA, F = 28.1, *p* < 0.0001, with a significant injury effect, ^**^p < 0.0001 for both injured vs. sham and injured vs. penumbra, *post-hoc* Tukey). We measured the rh123 levels a second time at 15 min post-injury to determine if modulation of electrical activity could facilitate recovery. We used difference images for the three drug treatment conditions of (i) control, (ii) TTX, and (iii) bicuculline to determine recovery (Figure 8C). Control neurons from both the injury zone and penumbra demonstrated a continued increase in mitochondrial depolarization as indicated by a small rise in rh123 levels. Drug treatments produced a differential effect, with silencing activity leading to a reduction in rh123 fluorescence that was not significantly different from uninjured controls, while promoting activity leading to an increase in rh123 fluorescence over untreated injured cultures and uninjured cultures (Figure 8D).

We then analyzed these same data to calculate the percent of cells with an increased rh123 signal and found a significant increase with injury and a significant decrease with TTX (Figure 8E) (two-way ANOVA, F = 5.51, *p* < 0.005, injured vs. sham, ^**^p < 0.005 and TTX vs. control, ^*^p < 0.05, *post-hoc* Tukey). Interestingly, within the injured category, there was a significant increase in rh123 when cultures were treated with BMB vs. control cultures (^*^p < 0.05, ANOVA with *post-hoc* Tukey). Full depolarization of the mitochondria with 0.2 μM FCCP treatment, a protonophoric uncoupler, revealed that the injured neurons had mitochondria which were maximally depolarized (two-way ANOVA, F = 5.39, *p* < 0.005 with a significant injury effect, ^**^p < 0.005 for injured vs. sham, *post-hoc* Tukey; Figures 8F,G). From these data, we conclude that silencing network activity is beneficial post-injury as it reduces mitochondrial depolarization. Conversely, increasing electrical activity with BMB is detrimental to the mitochondrial function of injured neurons. The amplitude of these changes were small but were detected within a very short time period of 15 min.

## Discussion

By providing a means to control the biophysical and biochemical environment, *in vitro* models have been critical for understanding the basic molecular mechanisms of TBI. In this study, we developed an injury model with high spatial and temporal resolution, allowing us to monitor individual cells as they are injured and then follow the propagation of that injury through a network of neurons and astrocytes. With the ability to injure specific neurons, the impact of regional injury within a network of cells could readily be investigated. Our findings demonstrate the highly localized injury to neuronal networks *in vitro* could initiate a response well beyond the initial area of mechanical injury, and this propagation is mediated primarily by ATP/purinergic signaling. Progressive injury to a network impairs both the structure and synchronization of neurons within the network, and both the initial integration and activity of a neuron within a network influence the primary response of the neuron to injury. The long-term response to these targeted injuries to neurons within a network showed that neuronal death is best predicted by a combination of the neuron's integration within the broader network, the initial mechanical severity of the injury, and the coordination of the neuron's activity with neighboring neurons in the network. Finally, our results indicate that modulating activity can mediate mitochondria function in the post-acute phase following microtrauma, with silencing activity with TTX as an effective means to restore normal mitochondria function soon after injury. Together, these data point to the important interplay between activity and a neuron's integration within a neuronal network and the role that mitochondria can play as a mediator between the initial response to trauma and the longer term structural changes to the network.

Our first observations that mechanical stimulation can provide a much broader response throughout neuronal networks are consistent with previous studies. Past work using cultured neurons on deformable membranes demonstrated that after application of a uniaxial or biaxial stretch to the center of the membranes, neurons in unstretched regions respond with a rise in intracellular calcium (Lusardi et al., 2004; Choo et al., 2013). This secondary signal indicates a transmission of the initial signal to the penumbra. In primary astrocytes, the application of a micropipette tip is sufficient to produce a radial intercellular calcium wave that extends well beyond the initial area of stimulation (Murphy et al., 1993). However, no previous study has provided insight into how the combination of smaller, randomly placed “microtraumas” to a living neural circuit could affect the initial and longer term function of the network. This may be especially important as the *in vivo* transfer of mechanical force during an impact that causes brain injury likely deforms some neurons more than others (Karami et al., 2009; Feng et al., 2013; Singh et al., 2015; Labus and Puttlitz, 2016; Yousefsani et al., 2018; Wu et al., 2019), and this heterogeneity of stress transfer may be an important factor in the acute functional response after trauma. Similar to the stretch model, we found that the local fluid pulse injury initiates a calcium wave that projects outwardly from the injury center to non-injured astrocytes in an ATP-dependent manner. Propagation to non-injured neurons was also observed but typically required higher levels of injury. Blocking both NMDAR and purinergic receptors was sufficient to significantly attenuate the latent response of elevated intracellular calcium in neurons well outside the area of mechanical injury. The inability of NMDAR antagonism to block the primary response suggests that mechanoactivated NMDA receptors (Singh et al., 2012) are not a primary mediator of the initial response. Similarly, blocking the mechanoactivation of sodium channels (Joshi et al., 2006; Beyder et al., 2010) is not a mediator either. We cannot discount the possibility that the microtrauma response may trigger calcium release from intracellular stores (Rzigalinski et al., 1998; Weber et al., 2001; Kakizawa et al., 2012), possibly through the release of glutamate from injured neurons (LaPlaca and Thibault, 1998). Moreover, given that ionotropic P2X receptors are remarkably permeable to calcium ions (Pankratov et al., 2009), these could be an additional mediator of the primary injury response within the injury zone. Similarly, metabotropic P2Y receptors may play a role by inducing the release of intracellular calcium through IP_3_-dependent mechanisms (Egan and Khakh, 2004; Pankratov et al., 2009). Interestingly, not all neurons in the uninjured regions displayed a latent response, so future studies to further understand the mechanism should also address cell to cell variability in terms of purinergic receptor expression.

The perturbations in intracellular calcium induced by the local injury model had immediate effects on the regional network activity and functional connectivity. Surprisingly, perturbations to the network did not extend beyond the injured site. These findings indicate that networked neurons *in vitro* form largely local connections, and the targeted injury of an individual or small cluster of neurons leads to a loss of synaptic inputs to only neighboring neurons. Therefore, the disruptions in structure should remain largely localized. There is evidence that distance-dependent network structures form *in vitro* (Schmeltzer et al., 2014; Singh et al., 2015; Nigam et al., 2016; Lonardoni et al., 2017) and also develop *in vivo* (Hasel et al., 2015; Vegué et al., 2017; Tumulty et al., 2020), implying that if one can successfully reduce the transmission of mechanical injury to the remote penumbra regions, the consequences of an injury are more likely to be minimized. This possibility is most likely to occur in cortical areas, where local communities of neurons are physically close to one another but form distinct network modules. A more challenging network structure is when brain areas are distant from each other, communicating to each other through the white matter tracts. Our results suggest that the primary mechanical response may be communicated, through neurons, to more distant sites with unknown effects. In the future, it would be of importance to examine the relative role of physical proximity vs. network proximity in affecting the overall changes in function after trauma.

Not surprisingly, the proximity and the severity of the local injury are key determinants in neuronal fate. Intriguingly, we also discovered that both the firing rate and the participation within a network after impact play significant roles in outcome, as both are directly correlated to higher survivability. Paradoxically, we found that silencing the electrical activity of a network over a time period of 6 h provided a much greater benefit in terms of survival at 6 h post-trauma. This result implies that active neurons express pathways that lend to their survival, but that active firing after an injury is not necessary for at least 6 h. Multiple studies have documented a process known as “acquired neuroprotection” (Hardingham and Bading, 2010). Ongoing synaptic activity promotes survival via NMDAR signaling as calcium influx signals to activate the neuroprotective transcription factors CREB and MEF2 (Mao et al., 1999; Bas-Orth and Bading, 2013) and to downregulate the apoptosis promoting factor PUMA (Lau and Bading, 2009). In fact, it was recognized years ago that rats in enriched environments are protected from seizure-induced neurodegeneration (Young et al., 1999). When action potential firing is induced before and after insult, neurons are protected from insults, including glutamate-induced excitotoxicity and trophic factor deprivation (Hardingham et al., 2002). Enhanced neuronal activity also enhances the thioredoxin anti-oxidant system and prevents stress-induced mitochondrial breakdown (Papadia et al., 2008; Lau and Bading, 2009). In contrast, when calcium enters the neuron at a high and sustained magnitude, the calcium overload can reach the mitochondria, opening the mitochondrial permeability transition pore (MPTP), after which the proton gradient is lost with the ultimate effect of ATP depletion and excessive ROS buildup. In our focused injury, part of the increase in [Ca_2+_] occurred via NMDAR signaling, but we identified other mechanisms, including extracellular ATP signaling, that play a role in rises to [Ca_2+_]_i_. Regardless of the exact mechanism, mitochondria acutely depolarize in response to excessive calcium influx, especially for calcium influx through NMDARs (Fujikawa et al., 2012). Our observation that depolarization begins to recover 15 min post-injury if the network activity is silenced with TTX treatment implies that activity occurring in the post-acute trauma phase may impair, rather than improve, network function. In support of this possibility, boosting neuronal activity with BMB proved to be detrimental to mitochondrial function. Overall, our data raise the following question—when normally “protective” synaptic signaling is superimposed onto a rise in calcium from a mechanical insult, does it convert the synaptic signaling to “harmful?” It is well-established that some components of synaptic signaling exist in delicate balance, where tipping the balancing point by activating different receptor subpopulations can transition physiological signaling into pathophysiological states, and vice versa. Our results suggest that mechanical trauma and electrical activity also exist in a similar delicate balance, where the background of trauma may convert a signal that is normally protective into a factor that promotes degeneration.

Neuronal activity is energetically expensive, with the brain accounting for 2% of the body weight but consuming 20% of energy at the resting metabolic rate. Calculations estimate that synaptic signaling uses 41% of the brain's ATP stores as energy with most expenditure at postsynaptic sites, as the restoration of ionic concentrations requires active pump mechanisms (Harris et al., 2012). With a rise in intracellular calcium, mitochondrial calcium also increases, and in turn, mitochondrial dehydrogenases are activated to promote the TCA cycle for energy production. Although a physiologic rise in calcium is well-tolerated by mitochondria, a pathological and sustained calcium signal can lead to dysfunction in traumatic injury (Lamade et al., 2019). In the case of synaptic activity with high demands for ATP, ATP could be depleted, and silencing neuronal activity for the 6 h period post-activity is beneficial. Since our analyses did not go beyond 6 h, we cannot rule out that loss of synaptic signaling for 6 h results in later-term cell death through delayed apoptotic mechanisms. However, highly active neural networks may be protected by signaling at the translational level for hours after electrical activity ceases (Bas-Orth and Bading, 2013). What remains for future studies is the determination of the proper interval of activity silencing for mitochondrial recovery without a loss of the neuroprotective effect of increasing electrical activity.

In closing, our study provides new data for understanding how complex physical inputs to a living microcircuit of neurons can lead to early and later changes in network structure. Our observations show that simply assuming a dose-dependent effect of mechanical trauma on a network does not accurately capture significant features of the network prior to trauma that may protect its structure nor does it provide an accurate perspective on changes to the structure of the network that follows complex traumatic insults. Moreover, our results show that modulating electrical activity in the post-acute period influences later, permanent changes to network structure. In particular, silencing of electrical activity for a period of time leads to protection against degradation of network structure. These results show the complex interrelationship among activity and network structure that occur for mechanically injured circuits and provides important information for rebuilding the structure of circuits after mechanical trauma.

## Data Availability Statement

The raw data supporting the conclusions of this article will be made available by the authors, without undue reservation.

## Ethics Statement

The animal study was reviewed and approved by the University of Pennsylvania Institutional Animal Care and Use Committee.

## Author Contributions

RM performed micro-mechanical assay, cell culture, experiments and analysis, interpreted the data, and wrote the manuscript. CvR produced micro-patterns, generated axon data, interpreted the data, and reviewed the manuscript. BF and DM interpreted the data and wrote the manuscript. All authors contributed to the article and approved the submitted version.

## Conflict of Interest

The authors declare that the research was conducted in the absence of any commercial or financial relationships that could be construed as a potential conflict of interest.
