# Supplementary material for: Regional Neurodegeneration in vitro: The Protective Role of Neural Activity
Source: Front Comput Neurosci. 2021 Mar 29;15:580107. doi: 10.3389/fncom.2021.580107 (PMC8039287; doi:10.3389/fncom.2021.580107)
Supplement: Supplementary file 1 [file Data_Sheet_1.PDF]

Supplementary Information 1

The force, F, applied to the micropost is related to its deflection δ as follows:

$$F = \frac{3EI}{L^3} \cdot \delta$$

Where E, I and L are the Young's modulus, moment of inertia, and length of the beam, respectively.

$$E = 2.5MPa = 2.5 \times 10^{-6} \frac{N}{\mu m^2}$$

$$I = \frac{\pi r^4}{4} = \frac{\pi (0.915)^4}{4} = .5505 \mu m^4$$

$$L = 8.3 \mu m$$

Under a uniform load, the deflection δ is a function of distance along the beam, computed as follows:

$$\delta(y) = F \cdot \frac{6L^2 - 4yL + y^2}{24EI}$$

So the end of the micropost has a deflection of:

$$\delta_{max} = F \cdot \frac{6L^2 - 4L^2 + L^2}{24EI}$$

Therefore:

$$\frac{F}{\delta_{max}} = \frac{8EI}{L^4} = \frac{8 \cdot (2.5 \times 10^{-6})(.5505)}{4745.8} = .00232 \times 10^{-6} \frac{N}{\mu m} = 2.32 \frac{nN}{\mu m}$$

And with an end-load, the applied force is related to the end deflection as:

$$\frac{F}{\delta_{max}} = \frac{3EI}{L^3} = \frac{3 \cdot (2.5 \times 10^{-6})(.5505)}{571.8} = .00722 \times 10^{-6} \frac{N}{\mu m} = 7.22 \frac{nN}{\mu m}$$
