# Supplementary material for: Regional Neurodegeneration in vitro: The Protective Role of Neural Activity
Source: Front Comput Neurosci. 2021 Mar 29;15:580107. doi: 10.3389/fncom.2021.580107 (PMC8039287; doi:10.3389/fncom.2021.580107)
Supplement: Supplementary file 2 [file Data_Sheet_2.PDF]

Supplementary Table 1

In silico primary model parameters

N *(number of neurons in network)*	60
iFraction *(inhibitory fraction)*	0.2
Inj *(injured fraction)*	[0; 0.13; 0.25; 0.5; 0.75]
Rec.AMPA *(number of AMPAR per synapse)*	80
Rec.NMDA *(number of NMDAR per synapse)*	20
Rec.GABA *(number of GABAR per synapse)*	12
tstep *(time step)*	0.002 seconds
g.AMPA *(AMPAR conductance)*	12 pS
g.NMDA *(NMDAR conductance)*	45 pS
g.GABA *(GABAR conductance)*	40 pS
E.AMPA *(reversal potential for AMPAR)*	0 mV
E.NMDA *(reversal potential for NMDAR)*	0 mV
E.GABA *(reversal potential for GABAR)*	60 mV
P.vt *(threshold voltage)*	30 mV
P.vp *(peak action potential voltage)*	15 mV
P.vrest *(resting voltage)*	64 mV
